# Role of Aldosterone and Mineralocorticoid Receptor in Cardiovascular Aging

**DOI:** 10.3389/fendo.2019.00584

**Published:** 2019-08-23

**Authors:** Stefania Gorini, Seung Kyum Kim, Marco Infante, Caterina Mammi, Sandro La Vignera, Andrea Fabbri, Iris Z. Jaffe, Massimiliano Caprio

**Affiliations:** ^1^Laboratory of Cardiovascular Endocrinology, IRCCS San Raffaele Pisana, Rome, Italy; ^2^Molecular Cardiology Research Institute, Tufts Medical Center, Boston, MA, United States; ^3^Department of Sports Science, Seoul National University of Science and Technology, Seoul, South Korea; ^4^Unit of Endocrinology and Metabolic Diseases, Department of Systems Medicine, CTO A. Alesini Hospital, ASL Roma 2, University of Rome Tor Vergata, Rome, Italy; ^5^Department of Clinical and Experimental Medicine, University of Catania, Catania, Italy; ^6^Department of Human Sciences and Promotion of the Quality of Life, San Raffaele Roma Open University, Rome, Italy

**Keywords:** endothelial dysfunction, mineralocorticoid receptor, vascular stiffness, RAAS, oxidative stress

## Abstract

The mineralocorticoid receptor (MR) was originally identified as a regulator of blood pressure, able to modulate renal sodium handling in response to its principal ligand aldosterone. MR is expressed in several extra-renal tissues, including the heart, vasculature, and adipose tissue. More recent studies have shown that extra-renal MR plays a relevant role in the control of cardiovascular and metabolic functions and has recently been implicated in the pathophysiology of aging. MR activation promotes vasoconstriction and acts as a potent pro-fibrotic agent in cardiovascular remodeling. Aging is associated with increased arterial stiffness and vascular tone, and modifications of arterial structure and function are responsible for these alterations. MR activation contributes to increase blood pressure with aging by regulating myogenic tone, vasoconstriction, and vascular oxidative stress. Importantly, aging represents an important contributor to the increased prevalence of cardiometabolic syndrome. In the elderly, dysregulation of MR signaling is associated with hypertension, obesity, and diabetes, representing an important cause of increased cardiovascular risk. Clinical use of MR antagonists is limited by the adverse effects induced by MR blockade in the kidney, raising the risk of hyperkalaemia in older patients with reduced renal function. Therefore, there is an unmet need for the enhanced understanding of the role of MR in aging and for development of novel specific MR antagonists in the context of cardiovascular rehabilitation in the elderly, in order to reduce relevant side effects.

## Introduction

The mineralocorticoid receptor (MR) is essential for blood pressure regulation and electrolyte and fluid homeostasis (1). MR activation by aldosterone evolved in response to dramatic changes in salt stress which occurred during the transition from aquatic to terrestrial life. Indeed, aldosterone first appeared in tetrapods (2) suggesting that the aldosterone-MR system was necessary to maintain ion balance during the transition from salt water to land. In mammals, the kidney maintains osmolarity and extracellular sodium concentration, as well as plasma volume and blood pressure (3). Aldosterone is produced by the adrenal glands and represents the most potent sodium-retaining hormone in mammals (4). Aldosterone secretion is stimulated under specific conditions, such as the increase in extracellular K^+^ ion concentrations or renin-angiotensin-aldosterone system (RAAS) activation in response to decreased vascular volume (5, 6). In addition to its well-established role in the kidney, MR is expressed in many non-epithelial tissues [i.e., adipose tissue (AT), heart, endothelial cells, vascular smooth muscle cells, brain, etc.]. In this context, abnormal MR activation contributes to relevant cardiovascular alterations by multiple mechanisms including enhanced oxidative stress, inflammation, fibrosis, vascular tone, and endothelial dysfunction (7). Importantly, MR displays a similar affinity for aldosterone and the physiological glucocorticoids (cortisol and corticosterone) (8). In epithelial tissues, as well as in endothelial cells (9) and smooth muscle cells (10), the enzyme 11b-hydroxysteroid dehydrogenase type 2 (11HSD2) is able to convert endogenous glucocorticoids to inactive metabolites (11), promoting MR activation by aldosterone. In non-epithelial tissues, where expression of 11HSD2 is virtually absent or extremely low, such as brain, cardiomyocytes, and adipose tissue, glucocorticoids represent the major ligand of the MR (12).

Aging is a universal and independent risk factor for cardiovascular diseases (CVD) including hypertension, coronary artery disease, congestive heart failure and stroke (13, 14). According to a report from the American Heart Association (15), the incidence and prevalence of CVD significantly increases with age, and about two-thirds of CVD deaths occur in people aged 75 and older. To date, the influence of aging on aldosterone secretion and function in humans is not well-characterized, and the specific role of MR activation in vascular aging still awaits demonstration. In animal models, MR contributes to rising blood pressure with aging by regulating myogenic tone, vasoconstriction, and vascular oxidative stress (16). Both oxidative stress (17) and inflammation (18) are key factors in the pathophysiology of age-related cardiovascular disease in humans. Telomeres length in white blood cells can be considered as a biomarker of oxidative stress and inflammation as their progressive attrition, due to cell replication, is increased by oxidative stress, and inflammation determines an increase in white blood cells turnover rate. White blood cells telomeres are shorter in CVD patients. Aldosterone is known to accelerate cardiovascular aging through processes that generate reactive oxygen species in several tissues as well as in white blood cells (19–22) and an inverse relationship between circulating aldosterone and white blood cells telomeres length has been documented in normotensive aged matched men (23).

Several recent studies showed that MR expression is increased in vascular smooth muscle cells of aged animals (24, 25). Molecular mechanisms have also been uncovered by which rising vascular smooth muscle cell MR contributes to increased vasoconstriction with aging (26). Moreover, recent histopathologic findings have clarified profound alterations of the zona glomerulosa in adrenal glands with aging, which together with the increased vascular MR expression, may provide a further explanation for enhanced cardiovascular risk in the elderly (27, 28).

In this review, we will focus on the age-related alterations of MR signaling and aldosterone secretion and will discuss their specific role in determining increased cardiovascular risk in the elderly. Finally, we will address the potential relevance of MR pharmacological antagonism in the elderly, in order to increase arterial compliance and prevent cardiovascular aging and the associated morbidity and mortality.

## RAAS Alterations With Aging

Several studies have shown that older healthy individuals display a reduction in renin-angiotensin-aldosterone system (RAAS) activity, with decreased plasma renin activity and lower levels of plasma renin and aldosterone under basal conditions (hyporeninaemic hypoaldosteronism) (29–33). The decline in plasma renin with age has been attributed to the effect of age-associated nephrosclerosis (34). Human studies with small sample sizes suggested that older individuals secrete less aldosterone than younger ones (35), resulting in a greater risk for hyperkalemia in older individuals (36), especially when coupled with the age-associated decline in glomerular filtration rate (GFR). Accordingly, renin synthesis and release gradually decrease in aging rats, resulting in lower levels of plasma renin (37). Moreover, older subjects also show an impaired ability to trigger adequate responses to RAAS stimuli, such as orthostatic hypotension, potassium infusion or sodium depletion (29, 38).

These age-related RAAS alterations have been attributed to different mechanisms occurring with aging, namely: (i) glomerulosclerosis and reduction in functional nephrons (39–41); (ii) impaired function of juxtaglomerular apparatus (e.g., reduced sympathetic stimulation of the juxtaglomerular apparatus) (39); (iii) reduced renal production of kallikrein (a serine protease contributing to the synthesis of active renin); and (iv) reduced angiotensinogen synthesis by the liver (39, 42).

Importantly, age-related changes in RAAS activity lead older individuals to reduced ability to reabsorb sodium and reduced renal tubular potassium excretion, resulting in higher risk for volume depletion, hyponatremia and/or hyperkalemia (36). Of note, the risk for hyperkalemia is further enhanced under specific conditions, such as metabolic acidosis, reduction in GFR, or use of drugs inhibiting renal tubular potassium excretion [i.e., angiotensin converting enzyme (ACE) inhibitors, angiotensin II (Ang II) type 1 (AT1) receptor antagonists, MR antagonists, non-steroidal anti-inflammatory drugs (43)].

Recent reports clarified the histopathological changes occurring in adrenal glomerulosa cells with aging (27). The development of specific antibodies against aldosterone synthase (CYP11B2—the enzyme required for the final step of aldosterone production) recently allowed the detection of non-neoplastic foci of CYP11B2-expressing cells in the adrenal, referred to as aldosterone-producing cell clusters (APCC), which are commonly observed in normal human adrenals. Interestingly, recent studies revealed that the classic continuous CYP11B2 expression pattern within adrenal zona glomerulosa is gradually lost with aging, whereas accumulation of APCC in adrenal glands is frequently observed with advancing age. A direct evidence that APCC autonomously secrete aldosterone still awaits demonstration; however, aging is characterized by the transition from a physiological aldosterone regulation to a pattern of renin-independent aldosterone secretion, which could be sustained by increased number in APCC (28), and may account, at least in part, for the increased cardiovascular risk observed in the elderly (27).

Finally, it has been also shown that aging is associated with a decline in 11HSD2 activity, which results in renin suppression and cortisol-mediated MR activation (44), thus providing another potential mechanism for enhanced MR activation with aging.

Together, previous studies from both humans and animals provide evidence of altered RAAS activity and secretion with aging, which play a pivotal role in pathogenesis of CVD.

## Role of the Mineralocorticoid Receptor in Vascular Dysfunction With Aging

Aging is associated with structural, mechanical and functional alterations in the vasculature that are characterized by augmented vasoconstriction, reduced elasticity and distensibility, vascular stiffening, and impaired endothelial function (14, 45). These aging-related vascular changes contribute to cardiovascular disease and may be reversible; therefore, elucidating the mechanisms driving vascular aging has substantial potential to identify new therapeutic targets to prevent or reverse vascular aging, thereby attenuating the high CVD burden in the rapidly growing elderly population.

In addition to the traditional role of renal MR in regulating blood pressure by promoting sodium retention in the kidney (46), accumulated data in the past two decades indicate that MR is also expressed in the vasculature, including the smooth muscle cells, that contribute to vascular structure and vasoconstriction, and the endothelial cells, that contribute to barrier function and inflammation and thrombosis when injured (9, 10, 26). Substantial evidence support that MR in vascular cells contributes to CVD [reviewed elsewhere (47, 48)]. Animal studies have demonstrated that treatment with MR antagonists ameliorates vascular remodeling and dysfunction in the setting of CVD risk factors, including aging, western diet-induced obesity and hypertension, without significantly altering blood pressure (49–52), suggesting direct effects of MR antagonism on the vasculature. In clinical studies, MR antagonist treatment reduced vascular stiffness in elderly patients particularly with hypertension (53, 54).

MR expression increases in the vasculature with aging. Krug et al. found that MR gene expression is higher in aortas from aged rat (30 months of age) than in aortas from adult rat (8 months of age), and that MR protein expression was increased with aging in isolated rat aortic smooth muscle cells (24). More recent studies have similarly shown increased MR gene expression in mouse mesenteric resistance arteries with aging (25). To investigate the specific role for vascular smooth muscle cells MR in age-related mechanical and functional changes in the vasculature with aging, mice with smooth muscle cell-specific deficiency of MR (SMC-MR-KO) have been generated (26). Using these mice, McCurley et al. found that the moderate rise in blood pressure with aging in mice is prevented in SMC-MR-KO mice, without defects in renal function. Compared to aged MR-intact mice, 12 month-old SMC-MR-KO mice also showed decreased myogenic tone, vasoconstriction, and voltage-gated calcium channel expression, and decreased oxidative stress both at baseline and in response to Ang II (26). These findings indicate a direct contribution of smooth muscle cells-MR to increased vasoconstriction, vessel tone, and oxidative stress in aging vessels, which may contribute to the inexorable rise in blood pressure with aging. Further exploration of the mechanism by unbiased global miRNA expression profiling in mouse aortas, identified microRNA (miR)-155 as the most down-regulated miRNA in the aging vasculature. Interestingly, such down-regulation was prevented in SMC-MR-KO mice (25). DuPont et al. further demonstrated that MR transcriptionally represses the miR-155 host gene promoter. Thus, the increase in vascular MR expression with aging was associated with repression of miR-155 and increased expression of miR-155 target genes including the L-type calcium channel *(LTCC)* subunit Cav1.2 and the Ang II type 1 receptor (*Agtr1*), which are known to contribute to vasoconstriction and vascular oxidative stress with aging. These aging effects were prevented in SMC-MR-KO mice further supporting this as a mechanism by which smooth muscle cells-MR contributes to increased vasoconstriction, vessel tone and oxidative stress during aging (25).

**Figure 1:**
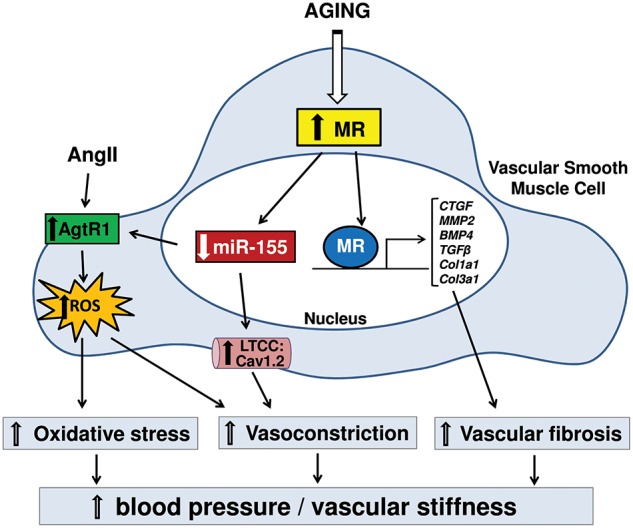
Diagram represents signaling for the contribution of mineralocorticoid receptor (MR) in smooth muscle cell (SMC) to vascular aging. Rises in SMC-MR expression with aging suppress miR-155, leading to the up-regulation of angiotensin type 1 receptors (AgtR1) and L-type calcium channels (LTCC), resulting in increased calcium influx and reactive oxygen species (ROS) production. This signaling causes enhanced vasoconstriction and oxidative stress. Also, increased MR in SMC with aging contributes to transcriptional activation of pro-fibrotic genes, leading to increased vascular fibrosis. These structural and functional changes with aging via MR in SMC result in hypertension and vascular stiffening. CTGF, connective tissue growth factor; MMP2, matrix metalloproteinase-2; BMP4, bone morphogenetic protein-4; TGFβ, transforming growth factor beta; Col1a1, collagen type-1 alpha-1; Col3a1, collagen type-3 alpha-1; Cav1.2, calcium channel; voltage-dependent; L type, alpha 1C subunit.

Smooth muscle cells-MR was also recently found to contribute to vascular structural changes with aging that determine vascular stiffening (16), a prominent consequence of aging in humans that correlates with risk of cardiovascular events (14, 45, 55). Although multiple CVD risk factors accelerate vascular stiffening, aging itself is associated with vascular stiffening that can occur independently and may even contribute to the development of other risk factors including hypertension (55–57). An important cause of vascular stiffness is excessive vascular fibrosis and reduced elasticity (45). Comparison of vascular stiffness with aging in MR-intact mice revealed increased aortic stiffness in 12 month- and 18 month-old mice compared to 3 month-old mice, along with increased fibrosis in aorta, carotid arteries and renal arterioles. These aging-associated increases in vascular stiffness and fibrosis were mitigated in SMC-MR-KO mice (16). Gene expression profiling in aortas revealed that MR deletion in smooth muscle cells induces a distinct anti-fibrotic gene profile in the aging vasculature, including downregulation of well-characterized pro-fibrotic genes such as connective tissue growth factor (CTGF), matrix metalloprotease-2 (MMP2), and bone morphogenetic protein-4 (BMP4) (16), that contribute to vascular fibrosis (14, 45). These findings indicate a role for smooth muscle cell-MR in vascular aging as a transcriptional regulator that activates pro-fibrotic genes with aging, consistent with prior studies showing that aldosterone activates pro-fibrotic genes in mouse vessels (58) and in human coronary artery smooth muscle cells (10). Moreover, long-term treatment of aged mice with MR antagonist prevented the progression of vascular stiffening, reduced vascular fibrosis and induced a similar anti-fibrotic gene signature as smooth muscle cell-MR gene deletion (16). A small cohort study in humans also showed that MR antagonism treatment for 1 month reduced fibrotic biomarkers in the serum from elderly patients compared to placebo treatment (16). Altogether, the available preclinical data reveal that MR expression in smooth muscle cells of the vasculature increases with aging and induces structural, mechanical, and functional changes in vessels that contribute to vascular stiffness and to rising blood pressure with age (Figure 1). Mechanistically, smooth muscle cell-MR contributes to functional and structural alterations of vessels with aging through the role of MR as a transcriptional regulator of genes associated with vascular tone, oxidative stress and fibrosis. Although larger and longer clinical studies in elderly humans are warranted, these findings support the potential benefits of MR antagonism to treat vascular aging and associated morbidity with aging.

To our knowledge, the specific role of MR in other vascular cells, such as endothelial cells, myeloid cells, fibroblasts, or perivascular adipose cells, has not been directly investigated in the setting of aging. However, studies have demonstrated that endothelial cell-specific MR deficiency or MR antagonists treatment in mice prevents hormone- or diet-induced increases in endothelial cell stiffness, oxidative stress, leukocyte adhesion and the associated decrease in nitric oxide (NO) production (59, 60), which are prominent features of age-related vascular dysfunction (48). In addition, although smooth muscle cell MR does not contribute to atherosclerosis (61), endothelial cell MR has recently been implicated in vascular inflammation in mouse models of atherosclerosis, specifically in males (62). Prior studies have also implicated MR expressed by myeloid cells in atherosclerosis, in vascular inflammation, fibrosis and remodeling as well as T-cell MR in hypertension (63–65). Thus, MR in other cells contributes to important vascular phenotypes that are known to be associated with vascular aging, supporting the need for future studies to investigate directly the roles for non-smooth muscle cells MR in vascular aging.

## Role of Mineralocorticoid Receptor in Myocardial Dysfunction With Aging

The aging heart is characterized by various functional and structural changes, partially resembling some of the features observed in animal models of increased MR activation (66), such as inflammation, oxidative stress, collagen accumulation and fibrotic remodeling (66–68). A growing body of evidence has suggested an important contribution of aldosterone and MR activation to cardiac remodeling and heart failure (69, 70). MR expression was first detected in cardiomyocytes and endothelial cells of atria and ventricles almost 30 years ago (71). In the myocardium, MR is also expressed in cell types other than cardiomyocytes, including coronary vasculature and macrophages (71, 72). Interestingly, experimental studies have shown that mice with cardiomyocyte-specific overexpression of MR display oxidative stress-mediated coronary endothelial dysfunction and increased expression of pro-fibrotic markers (e.g., CTGF) (67, 69, 73). Wilson et al. demonstrated that rats exposed to mineralocorticoids excess undergo a series of inflammatory and oxidative stress responses before the onset of myocardial hypertrophy or fibrosis (74). A recent publication by Kim et al. indicates that smooth muscle cell-MR deletion attenuates aging-associated increases in cardiac stiffness. The increase in cardiac systolic stiffness with aging correlated with the degree of aortic stiffness, suggesting that cardiac benefits of smooth muscle cell MR deletion in mice may be secondary to the prevention of vascular stiffening (16).

Macrophage MR has been also found to play a key role in mediating cardiac tissue remodeling, stimulating the pro-inflammatory macrophage M1-like phenotype (known as “classically activated” macrophages) and regulating the transcription of different inflammatory and pro-fibrotic markers, such as tumor necrosis factor α (TNFα) and transforming growth factor β1 (TGF-β1) (68, 75). MR is also expressed on T lymphocytes and its overactivation upregulates CD8+ cytotoxic T cells and T helper 17 (Th17) cells infiltrating in the heart. Other studies showed that MR antagonism decreases Th17 polarization and induces the T regulatory cells phenotype (76, 77). Interestingly, pharmacological MR antagonism decreased the accumulation and activation of CD4+ and CD8+ T cells in the murine heart and T cells specific MR-knockout mice displayed reduced cardiac hypertrophy, fibrosis, and dysfunction (78). Moreover, the MR selective antagonist eplerenone improved the adverse cardiac effects of aging in spontaneously hypertensive rats, reducing myocardial fibrosis and improving left ventricular diastolic function and coronary hemodynamics (79).

MR activation can also affect myocardial electrical function, potentially causing lethal cardiac arrhythmias associated with heart failure (70, 80). Gómez et al. demonstrated that the overstimulation of cardiac MR pathway leads to increased ryanodine receptor activity and long-lasting and broader spontaneous calcium sparks, which potentially predispose to arrhythmias (81). Another study has shown that transgenic mice with cardiac-selective overexpression of human MR exhibit a high rate of death due to ion channel remodeling (reduced outward K^+^ transient current, increased Ca^2+^ influx), which results in prolonged ventricular repolarization and fatal ventricular arrhythmias in absence of structural cardiac defects. Importantly, administration of spironolactone in pregnant mice was able to prevent embryonic and postnatal death in the offspring, suggesting that offspring lethality was highly related to MR overexpression and activation (82).

Atrial fibrillation is the most frequent cardiac arrythmias in the elderly population (83). Interestingly, Tsai et al. found that atrial MR expression is significantly higher in patients with atrial fibrillation compared with individuals with normal sinus rhythm. In the same study, aldosterone increased the expression of α-1G and−1H subunits of the T-type calcium channel in cultured murine HL-1 atrial myocytes, leading to increased T-type calcium current and calcium overload, which was attenuated by the mineralocorticoid antagonist spironolactone (84). Accordingly, although there is no evidence showing a direct role of MR dysfunction in aging causing atrial fibrillation, we can speculate that increased MR signaling in heart tissue, due to aging, could represent a causal link between aging and atrial fibrillation. Further studies are needed to directly explore this possibility.

In summary, accumulating data demonstrate that MR contributes to aging-associated myocardial dysfunction with cell type-dependent mechanisms revealed by animal studies, thus supporting the potential benefits of MR antagonism to treat cardiac dysfunction, especially in elderly population.

## Role of Mineralocorticoid Receptor in Endothelial Dysfunction and Inflammation With Aging

Very little is known about the role of aldosterone and MR activation in the vasculature in the context of healthy human aging. Healthy endothelial cells secrete vasodilator mediators which activate signaling pathways inducing smooth muscle cells to relax and leading to vasodilation (85). Nitric oxide (NO) is produced by healthy ECs after activation of endothelial nitric oxide synthase (eNOS). NO represents a major mediator of endothelial-dependent vasorelaxation (86, 87). In patients with cardiovascular risk factors, such as hypertension, obesity and diabetes, extensive data demonstrate that MR activation contributes to endothelial dysfunction, through impairment of vasodilation induced by the endothelium (22, 85, 88–91). In human coronary endothelial cells, MR regulates several genes involved in inflammation and oxidative stress (9, 10). It is known that MR activation in endothelial cells contributes to cardiac inflammation and remodeling by promoting the expression of vascular cell adhesion molecule 1 (VCAM1), as shown in animal models of hypertension (92). Moreover, aldosterone-mediated endothelial MR activation leads to the overexpression of the intracellular adhesion molecule-1 (ICAM-1), thereby enhancing leukocyte adhesion to coronary artery endothelial cells (9, 93). *In vivo*, MR in the endothelium contributes to ICAM-1 and E-selectin expression thereby contributing to leukocyte slow rolling and adhesion to the vasculature, a critical step in the process of inflammation (62).

Reactive oxygen species have also been suggested to mediate the detrimental effects of aldosterone in the vasculature through MR activation (94, 95). Arterial superoxide levels increase with aging, in part because of the excessive activity of NADPH oxidase. Increased oxidative stress leads to the inactivation of nitric oxide (96) and consequent arterial stiffness (97). Several studies showed that MR activates NADPH oxidase-dependent superoxide production (22, 90) and MR blockade decreases NADPH oxidase activity, reduces superoxide formation, and improves nitric oxide bioavailability (98). Importantly, the sensitivity of the MR to aldosterone is enhanced in arteries from aged and/or hypertensive humans (99). In animal models with enhanced cardiovascular risk, endothelial dysfunction is driven by aldosterone activation of endothelial cell-MR. Spironolactone significantly improved endothelial function in middle cerebral artery in a spontaneously hypertensive rat model (100). Moreover, pharmacological MR inhibition or selective deletion of MR in endothelial cells prevented impaired vasodilation in a model of diet-induced obesity (101) specifically in females (102). Finally, selective endothelial cell MR deletion in mice improved endothelial dysfunction upon a challenge of Ang II induced hypertension (103).

Only few clinical studies evaluated the effects of MR antagonists on arterial stiffness in hypertensive patients. In two different studies, eplerenone showed higher efficacy in reducing arterial stiffness than atenolol and a thiazide type diuretic (53, 104). On the other hand, a study comparing eplerenone and amlodipine showed that the aortic pulse wave velocity decreased similarly in both groups (105). Interestingly, in a randomized study conducted by Hwang et al. on healthy older adults free from overt cardiovascular disease, pharmacological inhibition of MR did not decrease oxidative stress nor lead to improved arterial stiffness and wave reflections. These findings suggest that MR may not substantially contribute to oxidative stress in healthy human aging in the absence of additional risk factors (106). The same authors also showed that acute inhibition of MR in healthy aged adults led to impairments in vascular endothelial function, suggesting that the MR may induce beneficial physiological actions in regulating eNOS activity and flow-mediated endothelium-dependent dilation in healthy aging (107). Vascular smooth muscle responsiveness to exogenous nitric oxide was not influenced by acute MR antagonism in this population. Similarly, acute MR antagonism did not affect systemic blood pressure or circulating and endothelial cell markers of oxidative stress and inflammation (107). Other studies demonstrated that MR deletion in endothelial cells does not inhibit endothelium-dependent relaxation in healthy aorta (101), mesentery and coronary arteries (103). Conversely, in subjects with CVD risk factors, endothelial dysfunction seems to be dependent on MR activation. In this regard, studies conducted in animal models suggest that the specific role of MR activation in endothelial function depends on endothelial health and integrity (102, 108). Thus, it can be speculated that MR activation determines the induction of a vasodilatory response in healthy endothelium, and a vasoconstriction response (potentially mediated by smooth muscle cell–MR) when the endothelium is stressed or damaged.

**Figure 2:**
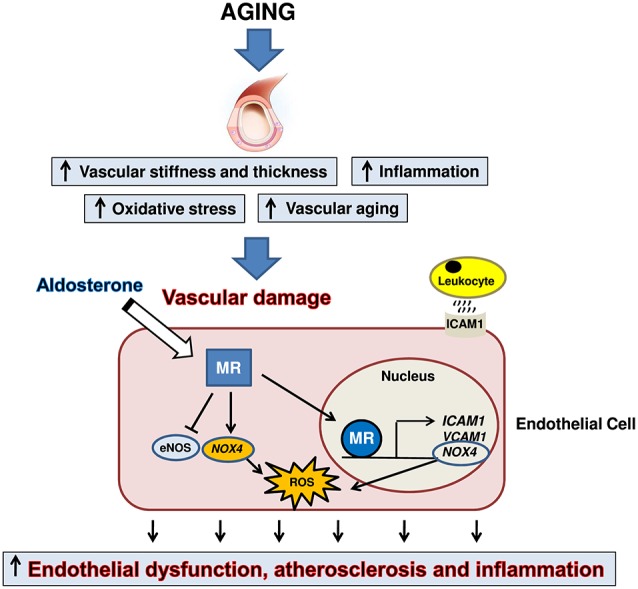
Aging is associated with vascular damage characterized by inflammation, vascular thickening, arterial stiffness and overproduction of reactive oxygen species (ROS). In this setting, mineralocorticoid receptor (MR) activation in endothelial cells can contribute to amplify cardiovascular adverse outcomes, exacerbating vascular stiffness through the induction of augmented ROS production, vascular inflammation and collagen deposition, finally leading to endothelial dysfunction and atherosclerosis. eNOS, endothelial nitric oxide synthase; NOX4, NADPH Oxidase 4; ICAM1, Intercellular Adhesion Molecule 1; VCAM1, vascular cell adhesion molecule 1.

Aging is associated with a progressive worsening of several physiological processes, leading to an increased risk of diseases, particularly at cardiovascular level (13, 14). Aging causes a pro-inflammatory state, remodeling of the vasculature, endothelial dysfunction and excessive production of reactive oxygen species (13, 14, 96, 109), mainly by increased expression and activity of NAD(P)H oxidase, which is not efficiently countered by antioxidant enzymes (110, 111). In the elderly, oxidative stress represents the most important cause of epigenetic modification (112) of the genes encoding for the antioxidant enzyme superoxide dismutase (113). In addition, the increased endoplasmic reticulum stress and proteasome activity elicits the process of unfolded protein response in vascular smooth muscle cells, monocytes, and endothelial cells (114). In this particular context of unhealthy aging, characterized by vascular damage, endothelial cell-MR activation can amplify cardiovascular adverse outcomes, exacerbating vascular stiffness through the induction of augmented reactive oxygen species production, collagen deposition, and vascular inflammation (9, 94, 95), resulting in altered vasodilation, endothelial dysfunction, and atherosclerosis (Figure 2).

In summary, a large body of evidence indicate that endothelial cell-MR is implicated in the pathological outcomes of cardiovascular risk factors, which are also highly associated with aging. Future studies are needed to determine if endothelial cell-MR plays a direct role in cardiovascular aging in animal models and humans.

## MR Antagonists in the Elderly: Clinical Studies

MR antagonists are largely used for the treatment of resistant hypertension and hearth failure (HF) (115), which represent highly prevalent diseases among older individuals (116, 117). In this context, several clinical trials demonstrated that cardiovascular morbidity and mortality are significantly reduced from the use of MR antagonists in moderate to severe heart failure with reduced ejection fraction (HFrEF) (118–120). In the double-blind Randomized Aldactone Evaluation Study (RALES), 1,663 patients with severe HFrEF and an average age of 65 years were randomly assigned to receive the MR antagonist spironolactone or placebo. After a mean follow-up period of 24 months, individuals from the spironolactone group showed a significant improvement in the symptoms of heart failure and a significant reduction in mortality, the latter attributed to the lower risk of death from cardiac causes (118). Thereafter, the Eplerenone Post-AMI Heart Failure Efficacy and Survival Study (EPHESUS) investigated the effects of the selective MR antagonist eplerenone on morbidity and mortality among 6,642 patients with an average age of 64 years and HFrEF following an acute myocardial infarction. After a mean follow-up period of 16 months, eplerenone significantly reduced the risk of death and hospitalization from cardiovascular causes and from any cause, as well as the rate of sudden death from cardiac causes (120). In contrast with these findings, the Treatment of Preserved Cardiac Function Heart Failure with an Aldosterone Antagonist trial (TOPCAT) found that spironolactone did not significantly reduce the rates of the primary composite outcome of death from cardiovascular causes, cardiac arrest, or hospitalization for heart failure in patients with heart failure with preserved ejection fraction (HFpEF) and a median age of 68.7 years (121). However, a *post-hoc* analysis has shown that spironolactone significantly reduced the TOPCAT primary outcome in patients with HFpEF from the Americas, suggesting that differences in demographic characteristics among recruited individuals may have represented a relevant bias of the study (122). On the other hand, a meta-analysis of seven randomized controlled trials evaluating the impact of MR antagonists on cardiovascular mortality and morbidity outcomes in patients with heart failure and/or left ventricular systolic dysfunction aged ≥65 years, did not confirm significant improvement in clinical outcomes among patients with HFpEF. However, the same study showed that MR antagonism improves clinical outcomes in selected cohorts of older patients with HFrEF (123). Another sub-analysis, which included 1,767 of the TOPCAT patients and was equally comprised of men and women, demonstrated that women with HFpEF had a significant reduction in cardiovascular and all-cause mortality with spironolactone, while men did not (124).

Interestingly, MR antagonists were also found to exert clinical benefit in patients with atrial fibrillation. In particular, a clinical trial on 164 patients aged ≥66 years with recurring atrial fibrillation showed that spironolactone, administered with β-blockers, was able to significantly prevent arrhythmic events, compared to spironolactone untreated patients (125). Recently, a retrospective cohort study of the contemporary ORBIT-AF (Outcomes Registry for Better Informed Treatment of Atrial Fibrillation) registry showed that the use of MR antagonists was not associated with reduced atrial fibrillation, but showed a trend toward lower risk of stroke, transient ischemic attack, or systemic embolism (126). However, the hypothesis that MR antagonists therapy may reduce residual stroke risk in patients with atrial fibrillation awaits demonstration in randomized clinical trials.

The recent 2018 ESC/ESH guidelines for the management of arterial hypertension now recommend that systolic blood pressure should be targeted to a range of 130–139 mmHg in older (>65 years) and very old (>80 years) patients (127). Importantly, recommended treatment of resistant hypertension considers the addition of low-dose spironolactone (up to 50 mg/day) to existing therapy also in the elderly population, where loop diuretics and alpha-blockers should be avoided due to their association with falls (128), extending the possibility of pharmacological MR antagonism in the aging hypertensive population.

In light of the significant cardiovascular benefits of MR antagonism in the aging population, their use in clinical setting is limited by the adverse effects induced by MR blockade on the kidney, such as hyperkalemia, particularly in older patients with reduced renal function and by their anti-androgenic properties (particularly exhibited by spironolactone) which can induce gynecomastia and erectile dysfunction in men (129, 130). Therefore, the current use of MR antagonists is restricted to patients with an estimated glomerular filtration rate >45 mL/min and a plasma potassium concentration of <4.5 mmol/L, in order to avoid the risk of hyperkalaemia (127). For such reasons, there is an unmet need for the development of more selective MR antagonist for heart and vasculature, in order to minimize the relevant side effects on non-cardiac tissues.

## Concluding Remarks

It is now clear that altered MR function is involved in the pathophysiology of endothelial dysfunction, atherosclerosis, oxidative stress, and cardiac remodeling. Altogether, these conditions are highly prevalent in the aging population and are deeply involved in the development of ischemic events and heart failure, common causes of morbidity and death in the elderly. Several recent studies demonstrated that aging is associated with important alterations in the aldosterone-MR system with changes in aldosterone production by the aging adrenal and increased MR responsiveness by the aging cardiovascular system. In accordance, clinical trials revealed the efficacy of MR antagonism in improving cardiovascular morbidity and decreasing mortality. The mechanisms involved in these cardiovascular benefits are complex and well beyond their well-known blood pressure lowering effects. It is now clear that systemic pharmacological antagonism produces direct effects in the vasculature and heart. However, MR pharmacological blockade in clinical practice has been limited by the risk of important adverse effects, such as hyperkalemia and renal dysfunction worsening, which is particularly frequent in aged individuals. Recently, a novel class of non-steroidal MR antagonist has been developed (131). Finerenone belongs to this group of molecules and its MR selectivity and affinity are higher compared to spironolactone and eplerenone. Due to these differences, finerenone may potentially reduce risk of both hyperkalaemia and renal impairment and, if so, may be safer to use in patients with heart failure affected by chronic renal dysfunction (132). Specifically, five phase II clinical trials demonstrated that finerenone is safe in patients with heart failure and concomitant chronic renal impairment and/or diabetes mellitus, and neither hyperkalemia nor reductions in kidney function were limiting factors to its use in over two thousand patients (133). Such favorable side effects profile is reached in the presence of similar clinical efficacy compared to other MR antagonists. Importantly, the addition of finerenone in patients with diabetic nephropathy resulted in improvement in the urinary albumin-creatinine ratio (134). ARTS-HF was the first clinical trial to compare finerenone with eplerenone, in patients with worsening HFrEF and chronic kidney disease and/or diabetes mellitus, with a mean age of 71.5 years. In such vulnerable population, finerenone reduced levels of NT-proBNP to a similar extent to that of eplerenone, but showed less changes in serum potassium from baseline to the end of the study in comparison to eplerenone (135). Importantly, finerenone at a dose of 10–20 mg demonstrated a nominally improved outcome of a composite clinical endpoint of death from any cause, CV hospitalizations, or emergency presentation for worsening heart failure (hazard ratio, HR: 0.56 [95% CI: 0.35–0.90]) compared to eplerenone in ARTS-HF. Moreover, preclinical studies showed that finerenone was able to potently block cardiac fibrosis and macrophages infiltration in a mouse model of isoproterenol-induced cardiac fibrosis, whereas eplerenone did not show significant effects (136). Nevertheless, phase III clinical trials will be crucial to further investigate the efficiency and safety of novel MR antagonists in the aging population, and studies on different subgroups of elderly people will help to identify new strategies to prevent cardiovascular aging, and to reduce the risk of end-organ damage related to MR activation (137).

## Author Contributions

SG and SK conceived and wrote the manuscript. MI wrote, in part, and revised the manuscript. CM prepared the figures and revised the manuscript. SL, AF, and IJ revised the manuscript. MC conceived, wrote, in part, and revised the manuscript.

### Conflict of Interest Statement

MC received research grants from Bayer AG. The remaining authors declare that the research was conducted in the absence of any commercial or financial relationships that could be construed as a potential conflict of interest.
